# Enhanced Slow-Wave EEG Activity and Thermoregulatory Impairment following the Inhibition of the Lateral Hypothalamus in the Rat

**DOI:** 10.1371/journal.pone.0112849

**Published:** 2014-11-14

**Authors:** Matteo Cerri, Flavia Del Vecchio, Marco Mastrotto, Marco Luppi, Davide Martelli, Emanuele Perez, Domenico Tupone, Giovanni Zamboni, Roberto Amici

**Affiliations:** Department of Biomedical and NeuroMotor Sciences, Alma Mater Studiorum - University of Bologna, Bologna, Italy; St. Joseph’s Hospital and Medical Center, United States of America

## Abstract

Neurons within the lateral hypothalamus (LH) are thought to be able to evoke behavioural responses that are coordinated with an adequate level of autonomic activity. Recently, the acute pharmacological inhibition of LH has been shown to depress wakefulness and promote NREM sleep, while suppressing REM sleep. These effects have been suggested to be the consequence of the inhibition of specific neuronal populations within the LH, i.e. the orexin and the MCH neurons, respectively. However, the interpretation of these results is limited by the lack of quantitative analysis of the electroencephalographic (EEG) activity that is critical for the assessment of NREM sleep quality and the presence of aborted NREM-to-REM sleep transitions. Furthermore, the lack of evaluation of the autonomic and thermoregulatory effects of the treatment does not exclude the possibility that the wake-sleep changes are merely the consequence of the autonomic, in particular thermoregulatory, changes that may follow the inhibition of LH neurons. In the present study, the EEG and autonomic/thermoregulatory effects of a prolonged LH inhibition provoked by the repeated local delivery of the GABA_A_ agonist muscimol were studied in rats kept at thermoneutral (24°C) and at a low (10°C) ambient temperature (Ta), a condition which is known to depress sleep occurrence. Here we show that: 1) at both Tas, LH inhibition promoted a peculiar and sustained bout of NREM sleep characterized by an enhancement of slow-wave activity with no NREM-to-REM sleep transitions; 2) LH inhibition caused a marked transitory decrease in brain temperature at Ta 10°C, but not at Ta 24°C, suggesting that sleep changes induced by LH inhibition at thermoneutrality are not caused by a thermoregulatory impairment. These changes are far different from those observed after the short-term selective inhibition of either orexin or MCH neurons, suggesting that other LH neurons are involved in sleep-wake modulation.

## Introduction

The lateral hypothalamus (LH) is a complex network of several different kinds of neurons involved in many functions [1]. LH neurons are apparently able to evoke a behavioural response that is integrated and coordinated with an adequate level of autonomic activity. In fact, the pharmacological activation of LH neurons has been shown to promote active behaviour and locomotion [2], and to coherently induce an increase in sympathetic outflow [3]. Both effects can be the consequence of the activation of a subpopulation of LH neurons producing orexin [4].

Recently, it has been shown that the inhibition of LH neurons prevented rats from producing rapid eye movement (REM) sleep [5]. The cause of this complete absence of REM sleep was suggested to be the inhibition of the activity of a subpopulation of LH neurons which produces melanin-concentrating hormone (MCH) [5]. However, optogenetic inhibition of MCH neurons did not produce a significant reduction in REM sleep duration [6]. This supports the hypothesis that REM-on GABAergic (non-MCHergic/non-orexinergic) neurons, which have also been observed in the LH, play a role in the regulation of REM sleep appearance [7]–[9]. Additionally, the inhibition of neurons within the LH depressed waking and evoked an extended period of non-REM (NREM) sleep [5]. These observations partially fit with the reported effects of either optogenetic [8], or DREADD (designer receptors exclusively activated by designer drugs) silencing of orexin neurons [10], and the administration of a dual orexin receptor antagonist [11]. However, in these three cases of selective inhibition of orexinergic activity, wakefulness was not comparably suppressed to the level shown after muscimol injection within the LH [5], suggesting that the role played by LH neurons in arousal levels cannot be entirely ascribed to the orexin neurons. Furthermore, in the latter studies the increase in NREM sleep was not nearly as great as that shown after the inhibition of the entire LH neuronal population, and REM sleep was still present.

While the outcome of LH neurons inhibition (reducing wakefulness) fits well with the observed effect of LH neurons activation (promoting wakefulness), changes in autonomic functions induced by such inhibition have not yet been investigated.

Of particular interest is the role played by LH neurons in thermoregulation control. Since the activation of LH neurons produces an increase in thermogenesis [3], it can be hypothesized that LH neurons inhibition may result in a state of hypothermia. While a modest reduction in brain temperature (core temperature around 36°C), such as that described after peripheral injection of CCK [12], can favour both NREM sleep and REM sleep occurrence, during either spontaneous torpor [13]–[16] or centrally-induced deep hypothermia (Core temperature 22°C, 24°C) [17], [18], REM sleep appearance was inhibited. Therefore, the possibility that LH inhibition may induce a state of marked hypothermia could provide an alternative explanation for the inhibition of REM sleep appearance described by Clement et al., 2012.

In order to evaluate whether sleep changes induced by LH neurons inhibition are the mere consequence of a reduced thermogenesis and not of the inhibition of specific wake-sleep (WS) related neural substrates within the LH, the present study aims to investigate the effects induced on both the WS cycle and thermoregulation by the pharmacological inhibition of LH neurons. Moreover, since Clement and co-workers used a single administration of the GABA_A_ agonist muscimol, leaving open the possibility that the relatively large vehicle volume and drug concentration might have caused an unwanted diffusion of the effects, we performed subsequent administrations of muscimol in small concentrations and volumes.

It is also critical to consider that neuronal inhibition can induce different effects according to the levels of neuronal activation preceding the inhibition. We therefore tested the effects of LH inhibition in different environmental conditions: at thermoneutrality, a condition that should not determine any specific activation of LH neurons, and during acute cold exposure, a condition that has been shown to increase the amount of wakefulness [19], and to induce a significant activation of LH neurons [20].

Here we show that the prolonged pharmacological inhibition of LH neurons almost abolished wakefulness and promoted a prolonged bout of NREM sleep characterized by an enhancement of slow-wave activity and by the absence of NREM-to-REM sleep transitions. A thermoregulatory impairment was observed when LH neurons were inhibited at an ambient temperature (Ta) of 10°C but not at Ta = 24°C.

Preliminary results of the experiments have been published in abstract form [21].

## Materials and Methods

### Ethical approval

The experiments were carried out with the approval of the Comitato Etico-Scientifico dell’Alma Mater Studiorum - University of Bologna (Ethical-Scientific Committee of the Alma Mater Studiorum - University of Bologna), in accordance with the European Union Directive (86/609/EEC) and under the supervision of the Central Veterinary Service of the Alma Mater Studiorum - University of Bologna and the National Health Authority. All efforts were made to minimize the number of animals used and their pain and distress.

### Surgical Procedures

Male CD Sprague-Dawley rats (n = 12, Charles River Inc, Lecco, Italy) were deeply anaesthetized through the injection of diazepam (Valium; F. Hoffmann-La Roche ltd, Basel, Switzerland, 5 mg/kg, intramuscular) followed by ketamine-HCl (Ketavet; Parke-Davis, Detroit, MI, USA, 100 mg/kg, intraperitoneal), and placed in a stereotaxic apparatus (David Kopf Instruments, Tujunga, CA, USA) with the incisor bar set in order to keep the bregma and lambda on the same horizontal plane. Animals were surgically implanted with: i) electrodes for EEG and nuchal electromyographic (EMG) recording; ii) a catheter placed into the femoral artery for the telemetric recording of arterial pressure (AP) (PA-C40, DataSciences International, St.Paul, MN, USA); iii) a thermistor (B10KA303N, Thermometrics Corporation, Northridge, CA, USA) mounted inside a stainless-steel needle (21 gauge) stereotaxically implanted above the left anterior hypothalamus to record the deep brain temperature (T_brain_); iv) two microinjection guide cannulas (C315G-SPC Plastics One Inc, Roanoke, VA, USA; internal cannula extension below guide: +3.5 mm), stereotaxically positioned in the left and the right LH. After surgery, animals received 20 ml/kg of saline subcutaneously and 0.25 ml of an antibiotic solution (penicillin G, 37500 IU; streptomycin-sulfate, 8750 IU) i.m. Animals recovered from surgery for at least one week, initially in their home cage and subsequently, for at least 3 days, in a Plexiglas cage with a stainless steel grid floor (wire diameter = 2 mm, inter-wire distance = 10 mm). The cage was positioned within a thermoregulated, sound-attenuated recording chamber where animals were kept throughout the experiment. The recording chamber was equipped with light and temperature controllers and acoustically insulated from the surroundings, so as to keep animals unaware of any activity outside the chamber. The recording chamber was located inside a Faraday-shielded room. Besides regular cage cleaning (at 9∶00 am every day), operators entered the Faraday-shielded room only during the microinjection procedure. During recovery from surgery, animals were kept at an ambient temperature (Ta) of 24°C±0.5°C and under a 12∶12 h light (L) - dark (D) cycle (light on at 09.00, 100 lux at cage level), and had free access to food and water. The recording chamber was also equipped with an infrared thermocamera (Thermovision A20, FLIR Systems, Boston, MA, USA) positioned under the stainless steel grid floor, to measure cutaneous temperature.

### Experimental Protocols

After recovery from surgery (1 week to 10 days), animals were divided into two experimental groups. Animals in group A (n = 5) were recorded for 6 consecutive days in the following conditions: i) day 1, baseline, Ta = 24°C; ii) day 2, inhibitor injections (GABA_A_ agonist muscimol, 1 mM, 100 nl, 1 bilateral injection/h starting at 11∶00 h and ending at 16∶00 h), Ta = 24°C; iii) day 3, recovery Ta = 24°C; iv) day 4, control, Ta = 24°C; v) day 5, saline vehicle injections (NaCl 0.9% w/v, 100 nl, 1 bilateral injection/h starting at 11∶00 h and ending at 16∶00 h), Ta = 24°C; vi) day 6, recovery, Ta = 24°C. Animals in group B (n = 7) were recorded for 6 consecutive days in the same conditions as those for group A with the exception that during both injection days (day 2 and day 5) animals were kept at Ta 10°C from 9∶00 h to 17∶00 h, the time period during which the injections were delivered.

### Microinjection procedures

The microinjection system consisted of a Hamilton 5 µl gastight syringe (Hamilton Company, Bonaduz, Switzerland) positioned in an infusion pump (MA 01746, Harvard Apparatus, Holliston, MA, USA; infusion rate 0.3 µl/min) and connected to the internal cannula through one meter of microdialysis FEP tubing (ID 0.12 mm OD 0.65 mm, Microbiotech/se AB, Stockholm, Sweden).

The cannula and the tube were filled with either muscimol (Tocris Bioscience, Bristol, UK) dissolved in vehicle solution or vehicle solution only (commercially available sterile-pyrogen free saline for parenteral injection (0.9%), S.A.L.F. Bergamo, Italy), while the syringe and the initial part of the tube were filled with coloured mineral oil. The insertion of the internal cannula into the guide cannula was performed manually by an operator by opening the lid of the recording chamber, gently inserting the internal cannula in the guide cannula and locking the two together. Care was also taken to avoid removing the animal from the cage during the insertion of the cannula. Once the internal cannula was inserted, the lid of the recording chamber was closed. The pump and the syringe were located outside the recording chamber.

All microinjection procedures were performed as follows. At 10∶55, the microinjecting cannula was inserted into the guide cannula. After closing the lid of the recording chamber, the first microinjection was performed. Ten minutes after the first injection, the lid of the recording chamber was opened again, the internal cannula extracted and inserted into the contralateral guide cannula. The lid was closed again, and the second microinjection was performed. After 10 more minutes, the recording chamber was opened and the cannula retrieved. This procedure was repeated for each of the 6 injections performed.

During each injection (average duration: 30 s±5 s), the volume injected (100 nl) was microscopically-assessed by the movement of the oil-liquid interface within the FEP tubing over a ruler. Compared to the single administration performed by Clement and co-workers [5] (muscimol, 1 µg/µl, 8.76 mM, in 300 nl), we thought that a sequence of injections (1 per hour) of a smaller volume (100 nl) and concentration (0.1 µg/µl, 1 mM) would reduce the possibilities of confounding effects induced by an unwanted diffusion to neuronal pools outside the LH.

### Histology

**Figure 1:**
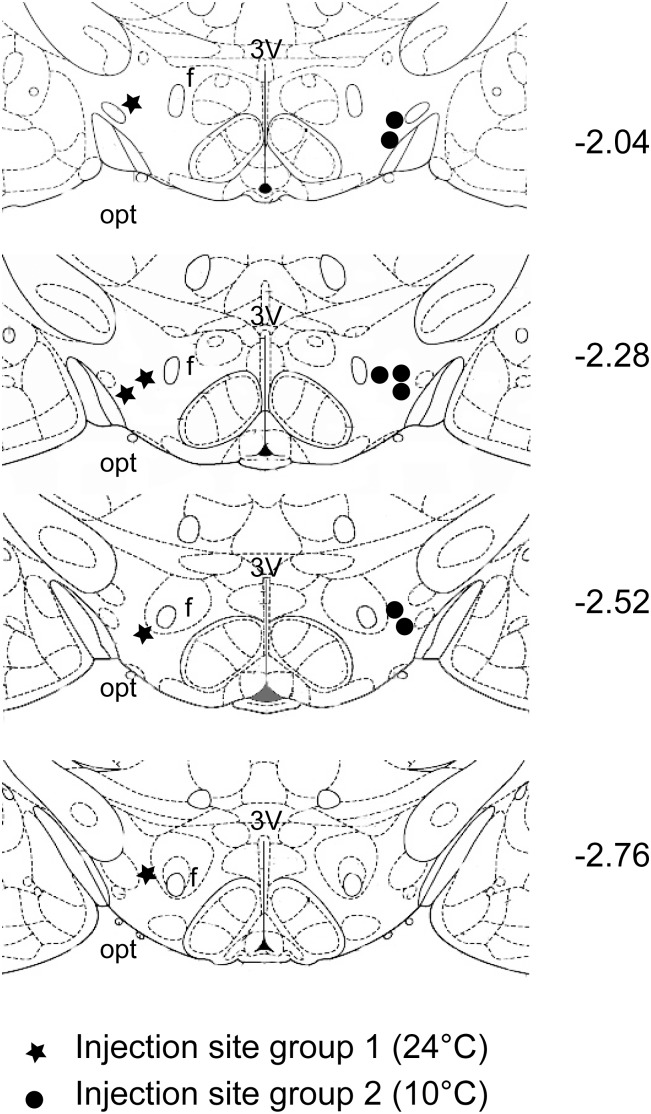
Injection Sites. The figure shows the location of the injection sites plotted on an atlas drawing [22]. Each injection was performed bilaterally but, for the sake of simplicity, is plotted monolaterally. Each slice refers to the antero-posterior distance in mm from Bregma, which is indicated on the right-hand side of each panel. The black stars, plotted on the left-hand side of each drawing, indicate the injection sites for group 1 animals (kept at an ambient temperature (Ta) of 24°C), while the black circles, plotted on the right-hand side of each drawing, indicate the injection sites for group 2 animals (exposed to Ta = 10°C during the injection period). 3V = third ventricle, f = fornex, opt = optic tract.

At the end of the experiment, the injection site was marked with 80 nl of Fast Green 2% dye. Rats were anaesthetized with ketamine as described above and transcardially perfused (4% w/vol paraformaldehyde). The brain was extracted and postfixed overnight with 4% paraformaldehyde and then cryoprotected (30% w/vol sucrose). The brain was then sliced coronally on a cryostat (60 µm) and sections containing a dye spot were plotted on an atlas drawing [22] (Figure 1).

### Signal Recording and Data Analysis

The EEG, EMG and T_brain_ signals were recorded by means of insulated copper wires connecting the headsocket to a swivel, amplified (Grass 7P511L, Astro-Med Inc, West Warwick (RI), USA), filtered (EEG: highpass 0.3 Hz, lowpass 30 Hz; EMG highpass 100 Hz, lowpass 1 KHz T_brain_ highpass 0.5 Hz), 12 bit digitalized (Micro MK 1401 II, CED, Cambridge, UK; acquisition rate: EEG: 1 KHz; EMG: 1 KHz; T_brain_: 100 Hz) and acquired on a digital hard drive. AP signal was telemetrically recorded, amplified and digitally stored on a hard drive (acquisition rate: 500 Hz). Heart rate (HR) was derived from AP peak detection.

EEG power spectrum was calculated from a 4-sec-long 1-sec-sliding window. EEG total power and power bands (Delta (0.5–4.5 Hz), Theta (5.0–9.0 Hz), Sigma (11.0–15.0 Hz)) were normalized to the mean value (100%) of the day 1 (control) recording. A full EEG spectrum from 0.25 to 20 Hz for NREM sleep and wakefulness was also calculated and normalized according to the average state specific spectrum of day 1. Sleep stages were visually scored by an operator (one-second resolution), using a script developed for Spike2 (sleepscore). Wakefulness, NREM sleep, and REM sleep were scored according to standard criteria based on EEG, EMG, and T_brain_ signals [19].

Digital images from the thermocamera were acquired at 1 frame/s and tail temperature (T_tail_) was measured in the medial portion of the tail by analyzing the thermographic record (Thermocam Researcher, FLIR systems, Boston, MA, USA). Variations of T_tail_ were analyzed comparing the 10-min average value recorded one hour before the first injection with the 10-min average value recorded 1 hour after the first injection for all experimental groups. Paired t-test was used to compare the pre-injection levels of T_tail_ with the post-injection values. Unpaired t-test was used to compare T_tail_ variation induced by muscimol injection with the saline-induced variation.

Values are reported as mean ± SEM. A two-way ANOVA (SPSS 21.0) with repeated measures on both factors was used for the statistical analysis of the results of the injection day (i.e. day 2 or day 5) and, with a different time resolution, of the baseline day (i.e. day 1 or day 4) together with the recovery day (i.e. day 3 or day 6). The modified t-test (t*) was used for both the pre-planned orthogonal and the pre-planned non-orthogonal contrasts [23], [24]. The α level of the non-orthogonal contrasts was adjusted using the sequential Bonferroni method [25].

For the analysis of the injection day, the Main Factors were defined as follows: i) the Factor “time” (which was considered for repeated measures) had 48 levels, corresponding to each 30-min interval of the whole 24-h period; ii) the Factor “Experimental Condition” (which was considered for repeated measures) had two levels (saline and muscimol). For each 30-min interval of the Factor “time”, data were compared by means of the following orthogonal contrast: saline *vs.* muscimol.

For the analysis of the baseline day and recovery day, the Main Factors were defined as follows: i) the Factor “time” (which was considered for repeated measures) had 4 levels, corresponding to the L and D periods of both days; ii) the Factor “Experimental Condition” (which was considered for repeated measures) had two levels (saline and muscimol). For each level of the “time” Factor, orthogonal contrasts were used to compare saline and muscimol results. For each level of the “Experimental Condition” Factor, the following pre-planned non-orthogonal contrasts were tested: recovery day_L *vs.* baseline day_L; recovery day_D *vs.* baseline day_D. The statistical analysis of the cumulative amount of wakefulness, NREM sleep, and REM sleep during the injection day was carried out with a *t*-test. The statistical analysis for T_tail_ was carried out comparing the 10 minutes T_tail_ average 1 hour before the beginning of the microinjection procedure with the 10 minutes T_tail_ average 1 hour later with a paired *t*-test. For all comparisons, statistical significance was set at p<0.05.

## Results

### Effects on sleep

**Figure 2:**
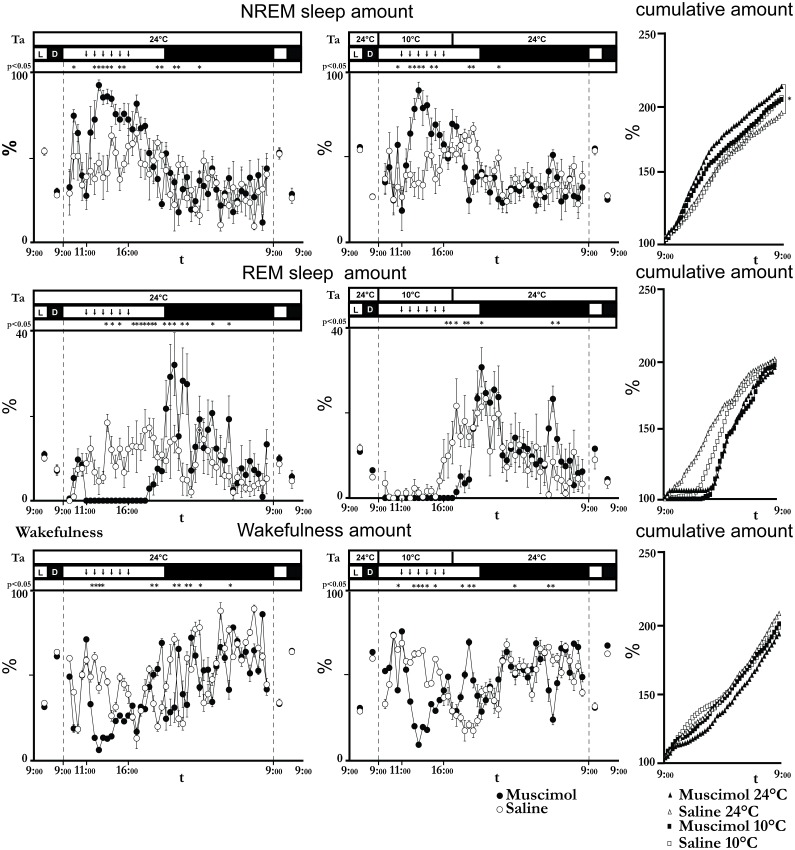
Sleep amount. The figure shows the time-course of the amount, expressed as the percentage of each epoch (12 h for Day 1, 3, 4, and 6; 30 min for day 2, and 5) of non-REM (NREM) sleep, REM sleep and wakefulness during the 6 experimental days (filled circles: days 1, 2 and 3; empty circles: days 4, 5 and 6) for group 1 animals (kept at an ambient temperature (Ta) of 24°C, left column) and group 2 animals (exposed to Ta = 10°C, from 9∶00 to 17∶00 of day 2, center column). The time resolution is 12 h for days 1, 3, 4, and 6 and 30 minutes for days 2 and 4. Vertical dashed lines divide consecutive experimental days. Each animal of each group was repeatedly injected with either the GABA_A_ agonist muscimol (day 2, filled circles, 100 nl, 1 mM, 1 injection/h bilaterally) or saline (day 4, empty circles, 100 nl, 0, 9%, 1 injection/h bilaterally). Ta, light (L)/dark (D) cycle and statistical significance are plotted above each panel. Each down-pointing arrow marks an injection. Data are shown as mean ± SEM. * = p<0.05. In the right-hand column the cumulative amount (expressed as the percentage of the respective total amount during the day preceding the injection day) of NREM sleep, REM sleep and wakefulness during each injection day is shown. * = p<0.05.

The 6-h inhibition of the LH neurons was characterized by a significant increase in the amount of NREM sleep at both Ta 24°C and Ta 10°C (Figure 2). At Ta 24°C, the amount of NREM sleep increased significantly during the injection period with a peak after the second injection (93.6±3.1%, t*_(192)_ = 4.43, p<0.05 compared to saline) and remained significantly higher compared to saline for the entire period of injections.

At Ta 10°C, the injections of muscimol induced a significant increase in the amount of NREM sleep that peaked after the third injection (90.5±4.9%, t*_(288)_ = 5.52, p<0.05 compared to saline). A significant negative peak in the amount of NREM sleep was observed between 20∶00 h and 21∶00 h and was associated with a peak in the amount of wakefulness (70.6±9.1%, t*_(288)_ = 4.15, p<0.05 compared to saline). On the recovery day after muscimol administration, no significant changes in the amount of NREM sleep were observed compared to the saline condition.

**Figure 3:**
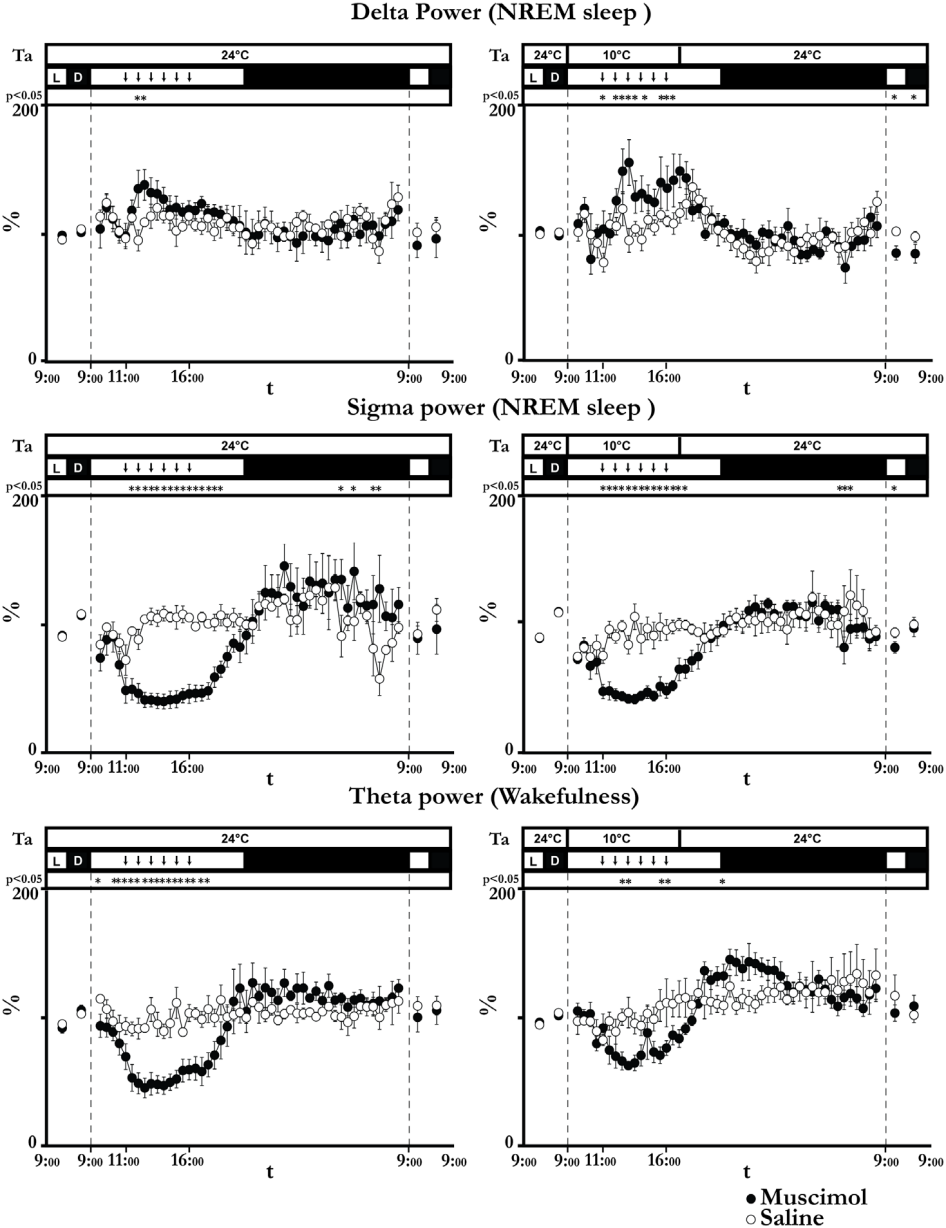
EEG power bands. The Figure shows the time-course of either Delta or Sigma power during non-REM (NREM) sleep and Theta power during wakefulness, during the 6 experimental days (filled circles: days 1, 2 and 3; empty circles: days 4, 5 and 6) for animals of group 1 (kept at an ambient temperature (Ta) of 24°C, left column) and for those of group 2 (exposed to Ta = 10°C from 9∶00 to 17∶00 of day 2, right column). Powers are normalized on the average EEG power of the day preceding the injection-day and expressed as percentages. The time resolution is 12 h for days 1, 3, 4, and 6 and 30 minutes for days 2 and 4. Vertical dashed lines divide consecutive experimental days. Each animal of each group was repeatedly injected with either the GABA_A_ agonist muscimol (day 2, filled circles, 100 nl, 1 mM, 1 injection/h bilaterally) or saline (day 4, empty circles, 100 nl, 0, 9%, 1 injection/h bilaterally). Vertical dashed lines divide consecutive experimental days. Ta, light (L)/dark (D) cycle and statistical significance are plotted above each panel. Each downward arrow marks an injection. Data are shown as mean ± SEM. * = p<0.05.

During the 6-h period of LH neuron inhibition, the Delta power in NREM sleep was significantly higher both at 24°C and at 10°C, while Sigma power in NREM sleep was significantly lower compared to the saline condition (Figure 3). At Ta 24°C, Delta power increased rapidly after the first muscimol injection, reaching a peak after the third injection (138.3±12.0%, t*_(192)_ = 2.59, p<0.05 compared to saline), and returned to a normal level before the end of the injection period. On the other hand, Sigma power was drastically reduced after the first muscimol injection and remained very low for the entire period of injections (nadir: 40.3±5.8%, t*_(192)_ = 5.48, p<0.05 compared to saline), slowly returning to normal at the end of the light period. At Ta 10°C, NREM sleep Delta power rapidly increased after the first muscimol injection and remained significantly elevated for the entire injection period (peak 155.7±18.0%, t*_(240)_ = 5.71, p<0.05 compared to saline). During the recovery day, Delta power showed a negative rebound; it was significantly lower compared to saline (Light period: 84.4±5.5%, t*_(20)_ = 4.22, p<0.05 compared to saline; dark period: 83.9±7.3%, t*_(20)_ = 3.32, p<0.05 compared to saline). Sigma power in NREM sleep was also drastically reduced, and remained significantly lower (nadir: 42.7±3.7%, t*_(288)_ = 6.37, p<0.05 compared to saline) for a few hours after the last injection.

**Figure 4:**
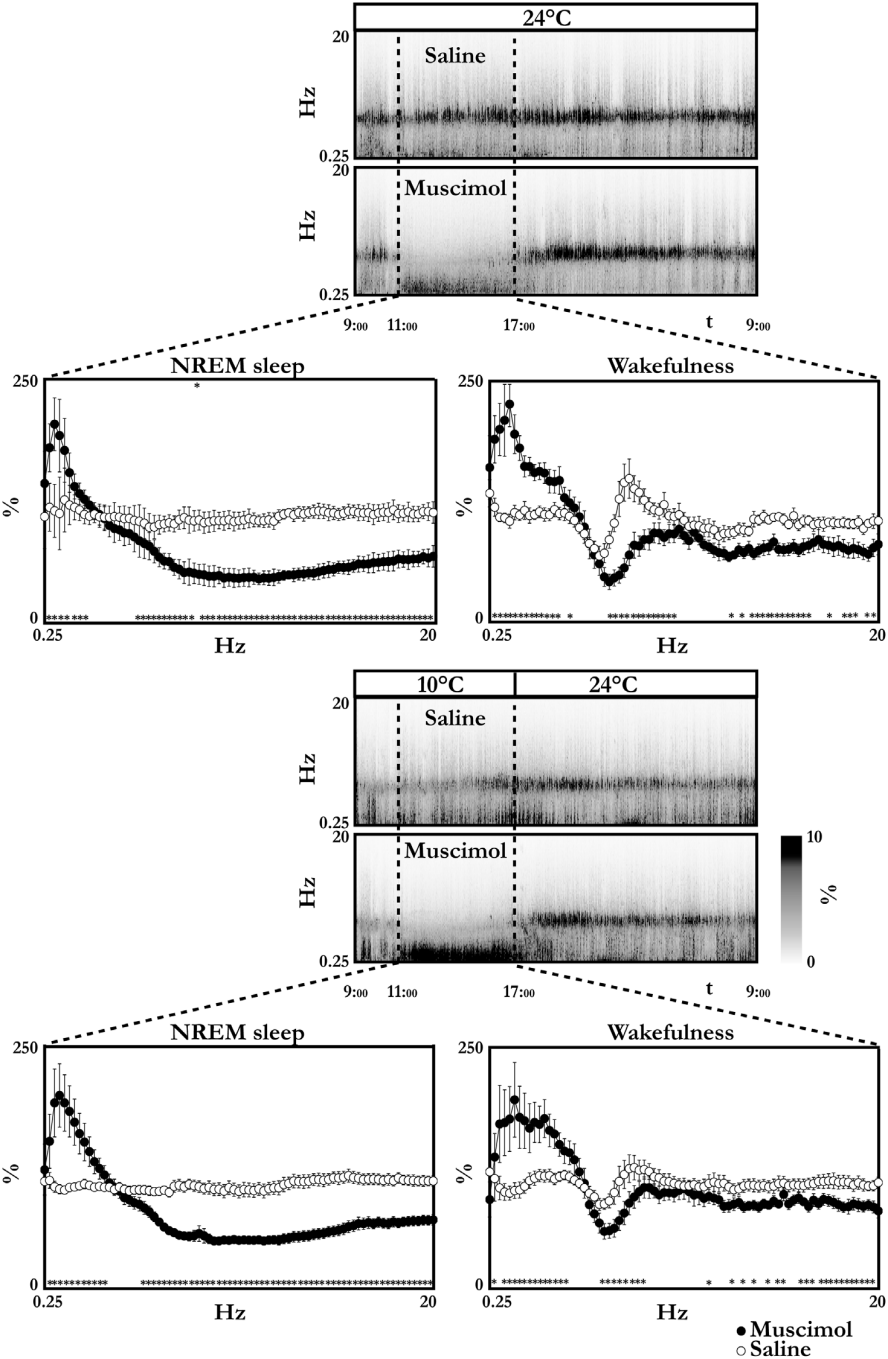
EEG spectra. The figure shows the time-course of the average EEG spectrum during the day of repeated injections of either the GABA_A_ agonist muscimol (100 nl, 1 mM, 1 injection/h bilaterally) or saline (100 nl, 0, 9%, 1 injection/h bilaterally) for group 1 animals (A and B) and for group 2 animals (E and F). EEG power is normalized on the average EEG power recorded during the day before the injection day and expressed as percentages. Ambient temperature is indicated above each panel. Vertical dashed lines indicate the injection period, from the first injection to 1 hour after the last injection. Panels C, D, G and H show the average EEG spectrum in non-REM sleep and in wakefulness during the injection period. Data are shown as mean ± SEM. * = p<0.05.

The EEG power spectrum in NREM sleep during the period of LH neuron inhibition showed a clear increase in the frequencies below 2 Hz at Ta 24°C and below 3 Hz at Ta 10°C, and a drastic decrease in all the spectral components above 7 Hz was observed at both Tas (Figure 4).

REM sleep was totally suppressed during the prolonged inhibition of the LH neurons and for a few hours afterwards, at both Ta 24°C and Ta 10°C (Figure 2). Acute exposure to Ta 10°C with saline injections also resulted in an immediate drastic decrease in REM sleep amount, but at around 15∶00 REM sleep appeared again. The amount of REM sleep lost during the injection period was fully recovered within the same day.

The changes in the amount of wakefulness induced by LH inhibition mirrored the effects on NREM sleep amount at both Ta 24°C and Ta 10°C (Figure 2). Theta power during wakefulness (Figure 3) was strongly decreased by the muscimol injections at both Ta 24°C (nadir: 47.4±6.8%, t*_(192)_ = 4.21, p<0.05 compared to saline) and Ta 10°C (nadir: 63.2±3.1%, t*_(288)_ = 2.13, p<0.05 compared to saline), returning to normal levels a few hours after the last injections. Animals exposed to 10°C showed an increase in Theta power during the night following the LH inhibition. No significant differences were observed during the recovery day.

The EEG power spectrum in wakefulness during the period of LH neuron inhibition showed a clear increase in the frequencies below 4 Hz at both Ta 24°C and Ta 10°C, and a drastic decrease in the frequency of the Theta band (Figure 4). Saline injections also produced a right-shift of the Theta region, which was around 1.5 Hz faster.

### Effects on autonomic variables

**Figure 5:**
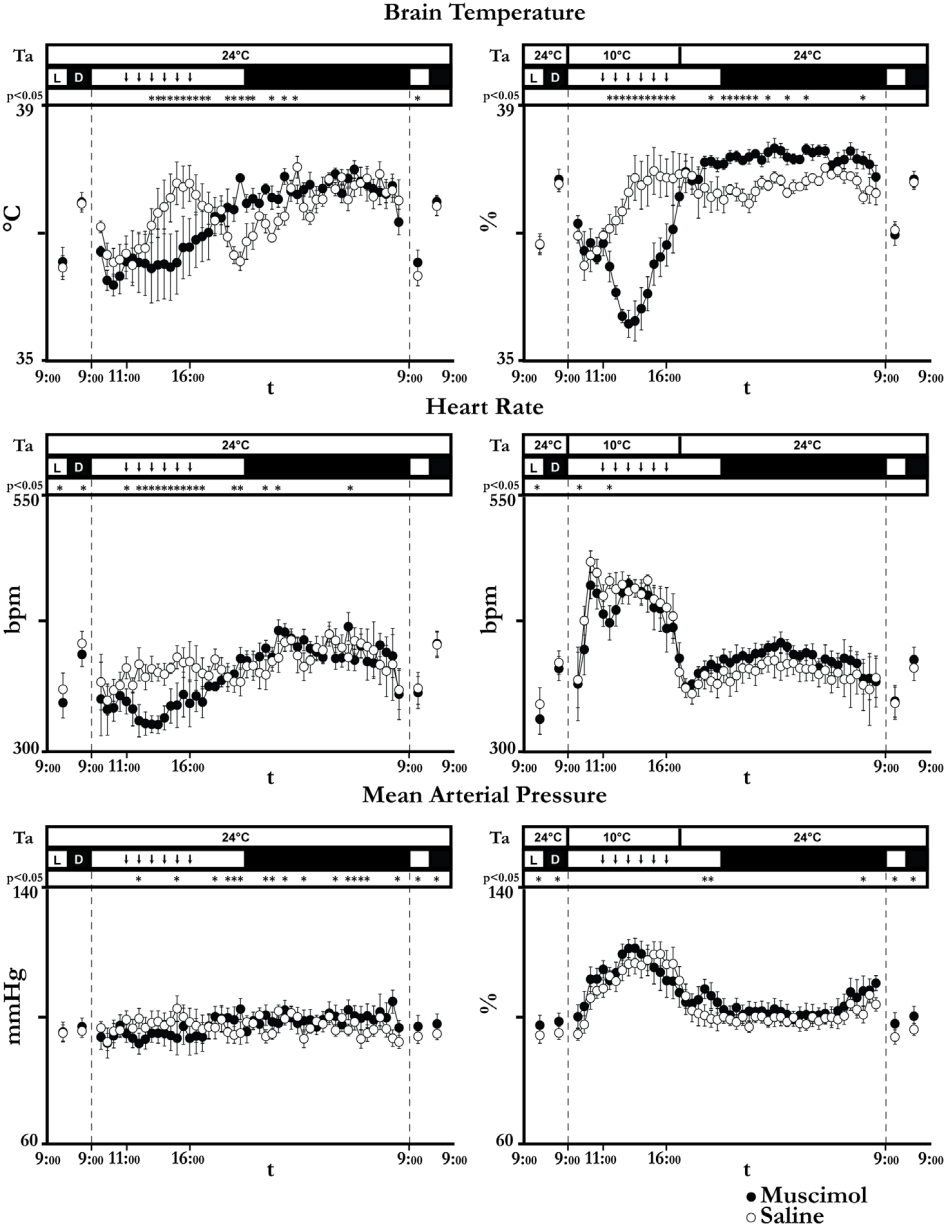
Autonomic parameters. The figure shows the time-course of brain temperature, heart rate and mean arterial pressure during the 6 experimental days (filled circles: days 1, 2 and 3; empty circles: days 4, 5 and 6) for animals of group 1 (kept at an ambient temperature (Ta) of 24°C, left column) and those of group 2 (exposed to Ta = 10°C from 9∶00 to 17∶00 of day 2, right column). The time resolution is 12 h for days 1, 3, 4, and 6 and 30 minutes for days 2 and 4. Vertical dashed lines divide consecutive experimental days. Each animal of each group was repeatedly injected with either the GABA_A_ agonist muscimol (day 2, filled circles, 100 nl, 1 mM, 1 injection/h bilaterally) or saline (day 4, empty circles, 100 nl, 0, 9%, 1 injection/h bilaterally). Vertical dashed lines divide consecutive experimental days. Ta, light (L)/dark (D) cycle and statistical significance are plotted above each panel. Each downward arrow marks an injection. Data are visualized as mean ± SEM. * = p<0.05.

**Figure 6:**
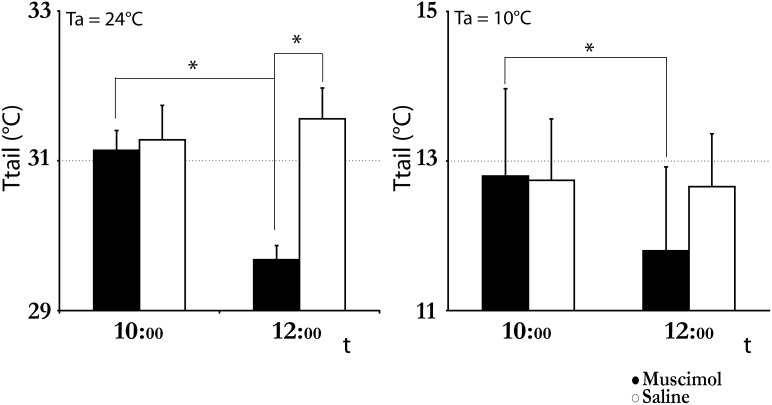
Tail temperature. The figure shows the average tail temperature measured from 9∶50 to 10∶00 (approximately 1 hour before the first injection) and from 11∶50 and 12∶00 (approximately 1 hour after the first injection of either muscimol (black bar) or saline (white bar)) at ambient temperature (Ta) = 24°C (on the left) or at Ta = 10°C (on the right). Data are shown as mean ± SEM. * = p<0.05.

At Ta 24°C, the repeated injection of saline produced a significant increase in T_brain_ compared to that observed during the injections of muscimol (peak: 37.8±0.3°C, t*_(192)_ = 5.20, p<0.05) (Figure 5). At Ta 10°C, saline injection still produced an increase in T_brain_, while muscimol injections evoked a decrease in T_brain_, that reached a nadir of 35.5±0.2°C (t*_(240)_ = 8.42, p<0.05 compared to saline) after 3 hours. T_brain_ returned rapidly towards physiological levels, but it remained significantly higher compared to the saline group for the rest of the day. No significant differences were observed in the recovery period. The decrease in T_brain_ was not caused by an increase in thermal dissipation, since the tail did not show any sign of vasodilation, but, rather, a modest but significant vasoconstriction (Figure 6), dropping from an average pre-injection value of 12.8±1.1°C to 11.8±1.1°C one hour after the first injection (t_(6)_ = 2.59, p<0.05). A significant reduction in T_tail_ was also observed at Ta 24°C, when T_tail_ dropped from an average pre-injection value of 31.1±0.3°C to 29.6±0.2°C one hour after the first injection (t_(4)_ = 8.48, p<0.05). The latter value was also significantly lower (p<0.05) compared to that observed one hour after the first saline injection (31.5±0.4°C; t_(5)_ = 3.55).

At Ta 24°C, HR was significantly higher following saline injections than following muscimol administration (Figure 5). The increase in HR induced by saline may be the result of the repeated microinjection procedure that, despite the care taken in trying to minimize the handling of the animal, may have disturbed the animal, resulting in a modest stress-induced hyperthermia. This effect was completely blocked by muscimol injection. At Ta 10°C, the acute cold exposure caused a rapid increase in HR that was only partially reversed by muscimol injections in a limited time window. No major effects on AP levels were observed following either muscimol or saline injections at both Tas.

## Discussion

The results of the present study show that, as previously described [5], in the rat kept at normal laboratory temperature (Ta, 24°C) the prolonged inhibition of LH neurons produced a pronounced increase in NREM sleep and a total suppression of REM sleep. These effects were not likely to be the mere consequence of changes in body temperature, since no decrease in T_brain_ was observed following muscimol injection in animals kept at Ta 24°C, suggesting that LH neurons do not play a role in the basic maintenance of body temperature in a thermoneutral environment.

A novel finding is that NREM sleep enhancement was characterized by an increase in Delta power, due to a large enhancement of slow wave activity (SWA), with almost no activity in the faster frequencies of the EEG spectrum. We categorized this state as NREM sleep, although its peculiar spectral EEG characteristics may call for an *ad hoc* denomination. Moreover, the results show that these effects were also produced when the inhibition of LH neurons occurred during acute cold exposure, a condition which is known to interfere with sleep processes [19].

The lack of relevant bodily thermal changes following muscimol injection at Ta 24°C suggests that the effects observed on either NREM sleep or REM sleep were the consequence of the inhibition of LH neurons specifically involved in the regulation of wake-sleep processes. Although the orexin and the MCH neurons within the LH may represent the best candidates [26], [27], a role for non-orexin/non-MCH neurons cannot be disregarded [9].

In fact, the features of the NREM sleep occurring after the inhibition of the entire population of neurons within the LH by muscimol substantially differ from those described after the selective inhibition of subpopulations of neurons in the same area, especially the orexin and the MCH neurons. As far as the orexin neurons are concerned, while their fast acute optogenetic inhibition was shown to be sufficient to rapidly induce SWA [28], and systemic pharmacological antagonism of orexin was shown to produce an increase in NREM sleep in several mammals [29], the 1-h optogenetically-mediated inhibition of these neurons in mice was effective in providing an increase in NREM sleep amount only when delivered during the dark period, but not during the light period of the LD cycle (i.e. the period in which the effect was observed in the present study) [8].

Also, while a pharmacological blockade of orexin receptors was not shown to induce significant changes in the NREM sleep EEG power spectrum in humans [30], [31], in the present study the spectral EEG characteristics of both NREM sleep and wakefulness during the period of muscimol injection appeared to be different from their physiological counterpart. Although the EEG trace during LH inhibition did not show evident abnormalities, the EEG power spectrum during NREM sleep, in particular, presented a large increase below 2 Hz and a drastic reduction in the faster frequency regions. In addition, the EEG spectrum of wakefulness was characterized by an increase in the power of the low-frequency region that was concomitant with a mild reduction in the power of the fast-frequency region. These findings, together with the observation that the activity of orexin neurons undergoes a circadian modulation that should be at its lowest level during our injection period [32], suggest that the increase in NREM sleep and in SWA induced by muscimol injections may be the results of the inhibition of a wider neuronal population within the LH rather than just the orexin neurons.

The inhibition of LH neurons at Ta 10°C induced a relevant hypothermia, confirming the role of LH neurons in thermoregulation and apparently providing an explanation for the suppression of REM sleep. However, the fact that after muscimol delivery at Ta 24°C brain temperature remained at baseline levels suggests that an impairment in thermogenesis was unlikely to be the cause of the absence of REM sleep. Interestingly, the almost complete suppression of Sigma power during NREM sleep indicates the absence of any attempt to enter REM sleep, since it is known that the NREM to REM sleep transition is marked by a strong increase in Sigma power [33].

The suppression of REM sleep may therefore be considered to be the consequence of the inhibition of the activity of MCH neurons [27]. However, the optogenetic inhibition of these neurons did not affect REM sleep duration [6], suggesting that other neurons in the area besides the MCH positive are involved in the regulation of REM sleep appearance. It can be suggested that this third population of neurons may be the population of GABA-positive/MCH-and-orexina-negative REM-on neurons [9]. These neurons may induce REM sleep by an inhibitory projection to the ventrolateral part of the periaqueductal gray and to the dorsal deep mesencephalic nucleus GABAergic REM-off neurons [27].

The REM sleep suppression caused by LH neurons inhibition may resemble the REM sleep suppression induced by an inflammatory state [34]. In both conditions the suppression of REM sleep is concomitant with an increase in NREM sleep and in Delta power, but LH inhibition does not induce any increase in brain temperature, as usually seen after cytokine injection [34]. In consideration of the fact that the increase in brain temperature during inflammatory response has been shown to be separable from the concomitant thermoregulatory effects [35], it is possible to hypothesize that the neuronal population within the LH may be one of the areas mediating the effects of cytokines on sleep.

Our results also show that LH neurons have a limited role in regulating the activity of the autonomic nervous system.

LH inhibition failed to reverse the increase in HR and MAP caused by cold exposure and had limited effects on HR at Ta 24°C, suggesting that LH neurons are not involved in the thermoregulatory modulation of cardiovascular parameters.

Thermoregulation was more largely affected than cardiovascular regulation by LH neurons inhibition. At Ta 10°C, T_brain_ decreased significantly during the delivery of the first three injections, but this decrease was quickly reversed, despite the fact that the LH neurons were still actively inhibited, suggesting the activation of a compensatory system. Since the decrease in T_brain_ was not caused by an increased thermal dissipation, it can only be explained by a reduced thermogenesis. It has been suggested that the orexin neurons within the LH are capable of amplifying an already active thermogenic drive, but have limited effects otherwise [4]. If this is the case, our data suggest that LH neurons, possibly the orexin neurons, are among the first responders that are activated to maintain a constant body temperature after cold exposure, potentiating the basic thermogenic drive, but they are not necessary for the maintenance of core temperature in a cold environment.

At Ta 24°C, no decrease in T_brain_ was observed, suggesting that LH neurons may not play a role in the basic maintenance of body temperature in a thermoneutral environment. Unexpectedly, an increase in T_brain_ was observed during the injection of saline. This can be explained by the repeated injection procedure that, despite every effort on behalf of the experimenters, may have produced some distress in the animal. An alternative explanation may lie in the fact that orexin neurons have been shown to be activated by a decrease in pH [36]. Even if the volume of the saline injection was very small, the possibility that a transient reduction in extracellular pH may have resulted in an activation of orexin neurons cannot be ruled out, although it appears unlikely. It is worth noting that, whatever the cause of the saline-related increase in T_brain_, the injection of muscimol suppressed it.

An increase in the vasoconstrictor tone in the tail blood vessels was induced by the muscimol injection both at Ta = 24°C and at Ta = 10 C°. The increased vasoconstriction may reveal the presence of a tonically-active inhibitory input originating in the LH and affecting other central areas involved in the regulation of cutaneous thermal conductance, such as the Preoptic Area [37] or the Raphe Pallidus [38]. Alternatively, it may be suggested that the reduction in the thermogenic drive induced by the inhibition of LH neurons immediately triggered a compensatory response that was sufficient to avoid hypothermia at Ta = 24°C but not at Ta = 10°C.

In conclusion, the results of our study show that: i) the acute inhibition of neurons within the LH induced a large increase in NREM sleep with enhanced SWA, and suppressed both REM sleep and the EEG activity characterizing the NREM to REM sleep transition; ii) these effects cannot be merely ascribed to the inhibition of either orexin or MCH neurons within the LH, suggesting a role for non-orexin/non-MCH neurons; iii) neurons located within the LH are involved in a first line of cold defense, but are not apparently essential for the maintenance of body temperature.

## Supporting Information

Dataset S1 **In the Dataset S1 the original data are reported.**
(XLS)

## References

[1] Berthoud HR, Munzberg H (2013) The lateral hypothalamus as integrator of metabolic and environmental needs: from electrical self-stimulation to opto-genetics. Physiol Behav 104: 29–39. 10.1016/j.physbeh.2011.04.051 PMC3131619 21549732

[2] Li FW, Deurveilher S, Semba K (2011) Behavioural and neuronal activation after microinjections of AMPA and NMDA into the perifornical lateral hypothalamus in rats. Behav Brain Res 224: 376–386. 21723327 10.1016/j.bbr.2011.06.021

[3] Cerri M, Morrison SF (2005) Activation of lateral hypothalamic neurons stimulates brown adipose tissue thermogenesis. Neuroscience 135: 627–638. 16125857 10.1016/j.neuroscience.2005.06.039

[4] Tupone D, Madden CJ, Cano G, Morrison SF (2011) An orexinergic projection from perifornical hypothalamus to raphe pallidus increases rat brown adipose tissue thermogenesis. J Neurosci 31: 15944–15955. 22049437 10.1523/JNEUROSCI.3909-11.2011 PMC3224674

[5] Clement O, Sapin E, Libourel PA, Arthaud S, Brischoux F, et al (2012) The lateral hypothalamic area controls paradoxical (REM) sleep by means of descending projections to brainstem GABAergic neurons. J Neurosci 32: 16763–16774. 23175830 10.1523/JNEUROSCI.1885-12.2012 PMC6621764

[6] Jego S, Glasgow SD, Herrera CG, Ekstrand M, Reed SJ, et al (2013) Optogenetic identification of a rapid eye movement sleep modulatory circuit in the hypothalamus. Nat Neurosci 16: 1637–1643. 24056699 10.1038/nn.3522 PMC4974078

[7] Hassani OK, Henny P, Lee MG, Jones BE (2010) GABAergic neurons intermingled with orexin and MCH neurons in the lateral hypothalamus discharge maximally during sleep. Eur J Neurosci 32: 448–457. 20597977 10.1111/j.1460-9568.2010.07295.x PMC2921479

[8] Tsunematsu T, Tabuchi S, Tanaka KF, Boyden ES, Tominaga M, et al (2013) Long-lasting silencing of orexin/hypocretin neurons using archaerhodopsin induces slow-wave sleep in mice. Behav Brain Res 255: 64–74. 23707248 10.1016/j.bbr.2013.05.021

[9] Sapin E, Berod A, Leger L, Herman PA, Luppi PH, et al (2013) A very large number of GABAergic neurons are activated in the tuberal hypothalamus during paradoxical (REM) sleep hypersomnia. PLoS One 5: e11766. 10.1371/journal.pone.0011766 PMC2909908 20668680

[10] Sasaki K, Suzuki M, Mieda M, Tsujino N, Roth B, et al (2011) Pharmacogenetic modulation of orexin neurons alters sleep/wakefulness states in mice. PLoS One 6: e20360. 21647372 10.1371/journal.pone.0020360 PMC3103553

[11] Betschart C, Hintermann S, Behnke D, Cotesta S, Fendt M, et al (2013) Identification of a novel series of orexin receptor antagonists with a distinct effect on sleep architecture for the treatment of insomnia. J Med Chem 56: 7590–7607. 23964859 10.1021/jm4007627

[12] Kapas L, Obal F Jr, Alfoldi P, Rubicsek G, Penke B, et al (1988) Effects of nocturnal intraperitoneal administration of cholecystokinin in rats: simultaneous increase in sleep, increase in EEG slow-wave activity, reduction of motor activity, suppression of eating, and decrease in brain temperature. Brain Res 438: 155–164. 3345423 10.1016/0006-8993(88)91334-0

[13] Walker JM, Garber A, Berger RJ, Heller HC (1979) Sleep and estivation (shallow torpor): continuous processes of energy conservation. Science 204: 1098–1100. 221974 10.1126/science.221974

[14] Harris DV, Walker JM, Berger RJ (1984) A Continuum of Slow-Wave Sleep and Shallow Torpor in the Pocket Mouse Perognathus longimembris. Physiological Zoology 57: 428–443.

[15] Krilowicz BL, Glotzbach SF, Heller HC (1988) Neuronal activity during sleep and complete bouts of hibernation. Am J Physiol 255: R1008–1019. 3202216 10.1152/ajpregu.1988.255.6.R1008

[16] Deboer T, Tobler I (1994) Sleep EEG after daily torpor in the Djungarian hamster: similarity to the effect of sleep deprivation. Neurosci Lett 166: 35–38. 8190354 10.1016/0304-3940(94)90834-6

[17] Cerri M, Mastrotto M, Tupone D, Martelli D, Luppi M, et al (2013) The inhibition of neurons in the central nervous pathways for thermoregulatory cold defense induces a suspended animation state in the rat. J Neurosci 33: 2984–2993. 23407956 10.1523/JNEUROSCI.3596-12.2013 PMC6619194

[18] Tupone D, Madden CJ, Morrison SF (2013) Central activation of the A1 adenosine receptor (A1AR) induces a hypothermic, torpor-like state in the rat. J Neurosci 33: 14512–14525. 24005302 10.1523/JNEUROSCI.1980-13.2013 PMC3761054

[19] Cerri M, Ocampo-Garces A, Amici R, Baracchi F, Capitani P, et al (2005) Cold exposure and sleep in the rat: effects on sleep architecture and the electroencephalogram. Sleep 28: 694–705. 16477956 10.1093/sleep/28.6.694

[20] Takahashi Y, Zhang W, Sameshima K, Kuroki C, Matsumoto A, et al (2013) Orexin neurons are indispensable for prostaglandin E2-induced fever and defence against environmental cooling in mice. J Physiol 591: 5623–5643. 23959674 10.1113/jphysiol.2013.261271 PMC3853500

[21] Del Vecchio F, Al-Jahmany A, Amici R, Cerri M, Luppi M, et al (2012) Effects on sleep of the inhibition of the lateral hypothalamus in the rat. J Sleep Res S358: 571.

[22] Paxinos G, Watson C (2007) The rat brain in stereotaxic coordinates. San Diego: Elsevier.

[23] Wallenstein S, Zucker CL, Fleiss JL (1980) Some statistical methods useful in circulation research. Circ Res 47: 1–9. 7379260 10.1161/01.res.47.1.1

[24] Winer BJ, Brown DR, Michels KM (1991) Statistical principles in experimental design. Boston: McGraw-Hill.

[25] Holm S (1979) A simple sequentially rejective multiple test procedure. Scand J Stat 6: 65–70.

[26] de Lecea L, Huerta R (2014) Hypocretin (orexin) regulation of sleep-to-wake transitions. Front Pharmacol 5: 16. 24575043 10.3389/fphar.2014.00016 PMC3921570

[27] Luppi PH, Peyron C, Fort P (2013) Role of MCH neurons in paradoxical (REM) sleep control. Sleep 36: 1775–1776. 24293748 10.5665/sleep.3192 PMC3825423

[28] Tsunematsu T, Kilduff TS, Boyden ES, Takahashi S, Tominaga M, et al (2011) Acute optogenetic silencing of orexin/hypocretin neurons induces slow-wave sleep in mice. J Neurosci 31: 10529–10539. 21775598 10.1523/JNEUROSCI.0784-11.2011 PMC3864636

[29] Brisbare-Roch C, Dingemanse J, Koberstein R, Hoever P, Aissaoui H, et al (2007) Promotion of sleep by targeting the orexin system in rats, dogs and humans. Nat Med 13: 150–155. 17259994 10.1038/nm1544

[30] Bettica P, Squassante L, Groeger JA, Gennery B, Winsky-Sommerer R, et al (2012) Differential effects of a dual orexin receptor antagonist (SB-649868) and zolpidem on sleep initiation and consolidation, SWS, REM sleep, and EEG power spectra in a model of situational insomnia. Neuropsychopharmacology 37: 1224–1233. 22237311 10.1038/npp.2011.310 PMC3306884

[31] Fox SV, Gotter AL, Tye SJ, Garson SL, Savitz AT, et al (2013) Quantitative Electroencephalography Within Sleep/Wake States Differentiates GABAA Modulators Eszopiclone and Zolpidem From Dual Orexin Receptor Antagonists in Rats. Neuropsychopharmacology 38: 2401–2408. 23722242 10.1038/npp.2013.139 PMC3799059

[32] Estabrooke IV, McCarthy MT, Ko E, Chou TC, Chemelli RM, et al (2001) Fos expression in orexin neurons varies with behavioral state. J Neurosci 21: 1656–1662. 11222656 10.1523/JNEUROSCI.21-05-01656.2001 PMC6762959

[33] Capitani P, Cerri M, Amici R, Baracchi F, Jones CA, et al (2005) Changes in EEG activity and hypothalamic temperature as indices for non-REM sleep to REM sleep transitions. Neurosci Lett 383: 182–187. 15936533 10.1016/j.neulet.2005.04.009

[34] Krueger JM, Johannsen L (1989) Bacterial products, cytokines and sleep. J Rheumatol Suppl 19: 52–57. 2691682

[35] Krueger JM, Takahashi S (1997) Thermoregulation and sleep. Closely linked but separable. Ann N Y Acad Sci 813: 281–286. 9100894 10.1111/j.1749-6632.1997.tb51706.x

[36] Williams RH, Jensen LT, Verkhratsky A, Fugger L, Burdakov D (2007) Control of hypothalamic orexin neurons by acid and CO2. Proc Natl Acad Sci U S A 104: 10685–10690. 17563364 10.1073/pnas.0702676104 PMC1965573

[37] Tanaka M, McKinley MJ, McAllen RM (2013) Role of an excitatory preoptic-raphe pathway in febrile vasoconstriction of the rat’s tail. Am J Physiol Regul Integr Comp Physiol 305: R1479–1489. 24133101 10.1152/ajpregu.00401.2013

[38] Cerri M, Zamboni G, Tupone D, Dentico D, Luppi M, et al (2010) Cutaneous vasodilation elicited by disinhibition of the caudal portion of the rostral ventromedial medulla of the free-behaving rat. Neuroscience 165: 984–995. 19895871 10.1016/j.neuroscience.2009.10.068

